# An Analytical Solution for Inverse Kinematics of SSRMS-Type Redundant Manipulators

**DOI:** 10.3390/s23125412

**Published:** 2023-06-07

**Authors:** Li Qin, Xiao Wei, Liangliang Lv, Liangliang Han, Guangqiang Fang

**Affiliations:** 1 Shanghai Institute of Aerospace System Engineering, Shanghai 201109, China; 2 Space Structure and Mechanism Technology Laboratory of China Aerospace Science and Technology Group Co., Ltd., Shanghai 201109, China; 3 Shanghai Institute of Satellite Equipment, Shanghai 200240, China

**Keywords:** inverse kinematics, redundant manipulator, analytical solution, algorithm singularity

## Abstract

Compared with non-redundant manipulators, the self-motion of 7-DOF redundant manipulators results in an infinite number of inverse kinematics solutions for a desired end-effector pose. This paper proposes an efficient and accurate analytical solution for inverse kinematics of SSRMS-type redundant manipulators. This solution is applicable to SRS-type manipulators with the same configuration. The proposed method involves introducing an alignment constraint to restrain the self-motion and to decompose the spatial inverse kinematics problem into three independent planar subproblems simultaneously. The resulting geometric equations depend on the part of the joint angles, respectively. These equations are then computed recursively and efficiently using the sequences of $\left(\theta_{1},\theta_{7}\right)$, $\left(\theta_{2},\theta_{6}\right)$, and $\left(\theta_{3},\theta_{4},\theta_{5}\right)$, generating up to sixteen sets of solutions for a given desired end-effector pose. Additionally, two complementary methods are proposed for overcoming the possible singular configuration and judging unsolvable poses. Finally, numerical simulations are conducted to investigate the performance of the proposed approach in terms of average calculation time, success rate, average position error, and the ability to plan a trajectory with singular configurations.

## 1. Introduction

**Figure 1:**
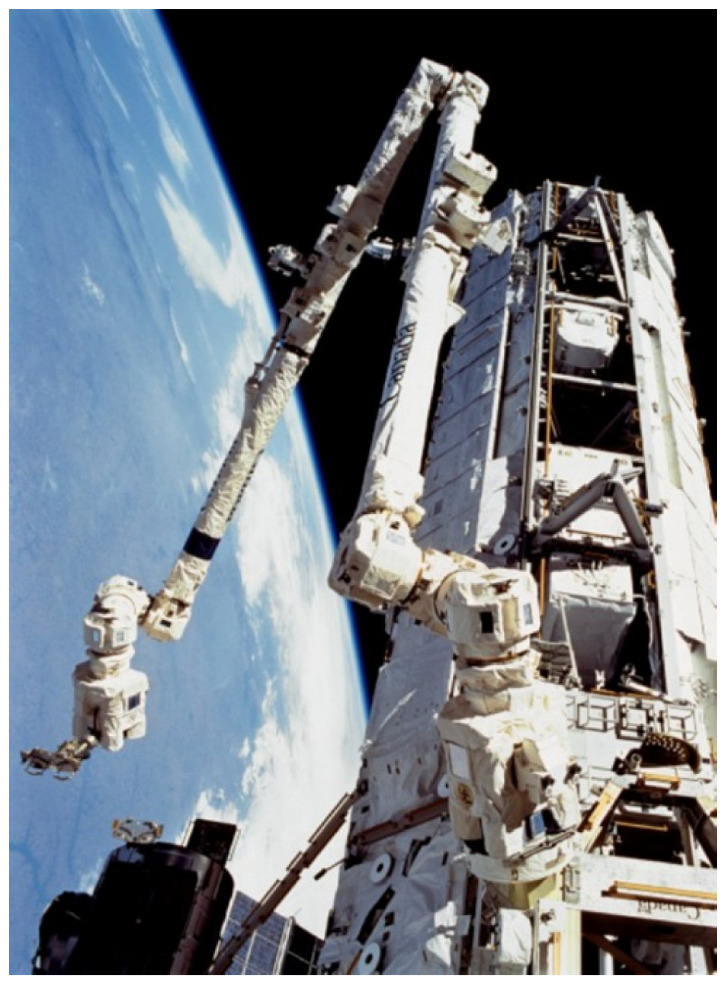
The Canadarm2 (SSRMS) [4].

**Figure 2:**
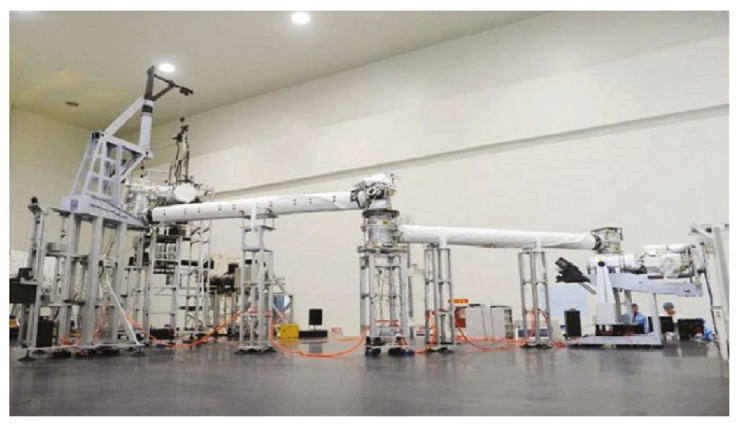
The EMM of the CSSRMS [7].

Seven degrees of freedom (7-DOF) serial manipulators have advantages over non-redundant manipulators in avoiding singularities, overcoming obstructed environments, preventing joint limits, and optimizing joint torques [1,2]. They are also a hot topic in a wide range of research areas, especially for on-orbit service that could be highlighted by their uses in the space station remote manipulator system (SSRMS) (Figure 1), the European robotic arm (ERA), and the special purpose dexterous manipulator (SPDM) for the International Space Station [3,4], as well as in the core module manipulator (cmm) and experimental module manipulator (EMM) (Figure 2) for the Chinese space station remote manipulator system (CSSRMS) [5,6,7], and the FREND [8], SPIDER [9], CAESAR [10], etc. However, the inverse kinematics (IK) problem for 7-DOF manipulators is under-constrained and is more complex to solve due to the presence of a redundant DOF, usually called ’self-motion’ [11]. Many analytical solutions have been proposed for this problem, including methods based on joint angle parameterization (i.e., the fixed-joint-angle method) and methods based on arm angle parameterization (i.e., the fixed-arm-angle method).

Those commonly used 7-DOF serial manipulators can be classified into two types: non-offset spherical-roll-spherical and offset manipulators that we call SRS-type manipulators and SSRMS-type manipulators, respectively, for convenience. At present, numerous studies focus on obtaining the analytical solutions for inverse kinematics of SRS-type manipulators. For example, Zu et al. [12] proposed a quadratic calculation method that adopted the joint angle parameterization method to obtain an accurate solution based on the approximate solution computed by the gradient projection method. Kreutz-delgado [13] parameterized the redundancy of SRS-type manipulators using a scalar variable (i.e., arm angle) that defines the angle between the arm plane and a reference plane, and proposed an arm angle parameterization method to solve the IK problem, which has an obvious physical significance and is suitable for controlling the manipulator configuration. Shimizu et al. [14] investigated the relations between the arm angle and the joint angles, and used the arm angle parameter to obtain all of the feasible IK solutions of SRS-type redundant manipulators with joint limitation constraints. Zhou et al. [15] proposed a position-based approach to obtain an analytical IK solution for SRS-type space manipulators with joint and attitude constraints and optimized the arm angle to minimize the base disturbance. Oh et al. [16] calculated the feasible range of the arm angle for avoiding self-collision and overcoming joint limits. However, the above methods based on the arm angle parameterization encounter singularity problems when the chosen reference unit vector is parallel to the vector from the shoulder to the wrist, Xu et al. [17] proposed a dual arm angle parameterization method to overcome this shortcoming.

Furthermore, some efforts based on the joint angle parameterization method were made to solve the IK problem of SSRMS-type manipulators. Crane et al. [18] reconstructed the SSRMS into a new 6-DOF sub-chain after parameterizing the joint angle $\theta_{1}$, $\theta_{2}$, or $\theta_{7}$, and obtained eight solutions for each case using spatial geometric projections. Xu et al. [19] obtained eight IK solutions for the SSRMS using the joint angle parameterization method, where $\theta_{1}$, $\theta_{2}$, $\theta_{6}$, or $\theta_{7}$ were selected as redundant parameters. Luo et al. [20] obtained an analytical IK solution for a 7-DOF manipulator with offset at the shoulder and wrist by selecting the joint angle $\theta_{1}$ or $\theta_{2}$ as redundancy parameters and proposed a method to determine the appropriate redundant joint.

Compared with SRS-type manipulators, SSRMS-type manipulators do not have the same definition of ‘arm angle’, thus the aforementioned methods based on arm angle parameterization are not suitable for SSRMS-type manipulators. Therefore, some scholars have simplified the SSRMS-type manipulator to the corresponding SRS-type manipulator and then used the relationship between them to obtain the analytical IK solutions. For example, Yu et al. [21] replaced the offset wrist joint with a virtual spherical wrist, and Xu et al. [19] took the place of the offset shoulder and offset wrist joints of SSRMS with two spherical wrists, respectively, and both of them obtained the IK solutions using the arm-angle-fixed method. Ma et al. [22] established a semi-analytical IK solution for the EMM, in which an associated simplified SRS-type manipulator was used to calculate the approximate solution, and the error was corrected using a numerical approach. Yang et al. [2] obtained the corresponding simplified SRS manipulator by setting the shoulder offset and wrist offset of the SSRMS manipulator to zero and solved the IK problem using an iterative algorithm based on the aforementioned dual arm angle parameterization method.

In addition, considering that the arm angle is dynamically defined and is difficult to determine intuitively, Gong et al. [23] defined a similar parameter ’elbow angle’ to constrain the redundancy of the SRS-type manipulators and found an analytical IK solution. Nammoto and Kosuge [24] presented an analytical IK solution for 7-DOF manipulators with an offset between joints 1 and 2, defined a variable ’offset angle’ to specify the self-motion of the manipulator, and used geometric information to obtain 16 sets of solutions. Jin et al. [25] proposed a hybrid of analytical and numerical IK solutions for SSRMS-type manipulators that used the elbow orientation as additional kinematic constraints to solve the IK problem and determined the optimal elbow orientation using the particle swarm algorithm.

In summary, in all of the above studies, it is central to add additional kinematic constraints, such as joint angle, arm angle, elbow angle, elbow orientation, etc., to determine the self-motion of the manipulator and to obtain the finite IK solutions that need to be specified intuitively according to the task, or determined by another numerical algorithm. This paper proposes an efficient and accurate analytical method to solve the IK problem of the SSRMS-type manipulators that introduces an alignment constraint to restrain the self-motion and to decompose the spatial inverse kinematics problem into three independent planar subproblems simultaneously. The proposed method deals with the SSRMS-type manipulator directly instead of with a simplified SRS-type manipulator, and results in explicit equations of the joint angles expressed by the desired end-effector pose matrix. The resulting geometric equations are computed recursively and efficiently and generate up to sixteen sets of solutions for a given desired end-effector pose. In this context, this paper is organized in the following manner. Following the introduction, Section 2 establishes a forward kinematic model of the SSRMS-type manipulator and finds an analytical IK solution based on the introduced alignment constraint between the axes of the second and sixth joints. In Section 3, the singularity of the proposed approach is figured out and then solved using a complementary method, and unsolvable desired poses are discussed. In Section 4, the performance of this method is verified using numerical simulations and using the SSRMS manipulator as an example. Finally, conclusions are given in Section 5.

## 2. Inverse Kinematics for SSRMS-Type Manipulators

### 2.1. Configuration and DH Model of the SSRMS

**Figure 3:**
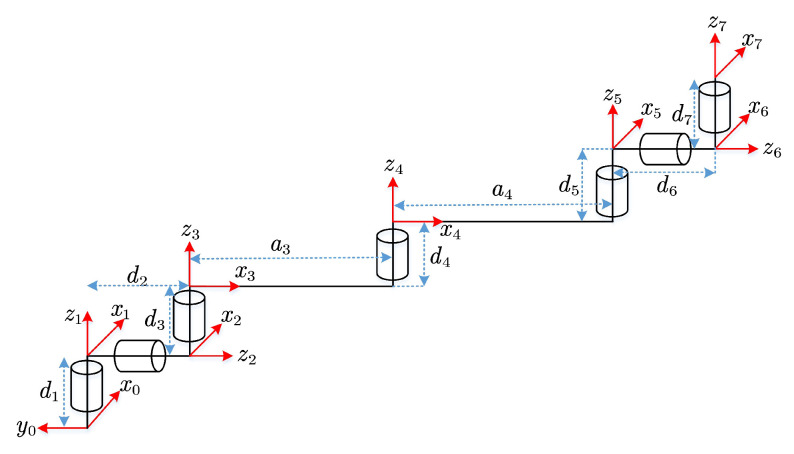
The initial configuration and DH frames of the SSRMS-type manipulator.

The joints of the SSRMS are arranged as (R-Y-P)-P-(P-Y-R), where each of the three joints of the shoulder or wrist has a link offset so that their axes do not intersect at one point, while the axes of the third, fourth, and fifth joints are parallel to each other [4]. The initial configuration and DH frames of the SSRMS-type manipulator are shown in Figure 3, where $\sum_{i}\left(i=0,1,\ldots ,7\right)$ are the coordinate systems of the manipulator. The modified DH parameters [26] are presented in Table 1, in which the shoulder offset $d_{2}$, elbow offset $d_{4}$, and wrist offset $d_{6}$ are nonzero. To visualize the geometric relationships when obtaining the inverse kinematics solution later, we introduced five auxiliary coordinate systems $\sum_{i^{\prime }}\left(i=2,3,\ldots ,6\right)$ that coincide with the corresponding $\sum_{i}$ in the initial configuration. It is noteworthy that $\sum_{i}$ is fixed on the link i−1 so $\theta_{i}$ is defined with the same measurement angle from $x_{i^{\prime }}$ (or $y_{i^{\prime }}$) to $x_{i}$ (or $y_{i}$) with respect to $z_{i}$.

### 2.2. Forward Kinematics

Based on the DH notation, the 4 × 4 homogeneous transformation matrix ${}^{i-1}T_{i}$(i=1,2,…,7) between the coordinate system $\sum_{i-1}$ and the coordinate system $\sum_{i}$ can be expressed as follows

(1) $${}^{i-1}T_{i}=Rot_{x,\alpha_{i-1}}Trans_{x,a_{i-1}}Rot_{z,\theta_{i}}Trans_{z,d_{i}}$$

(2) $${}^{i}T_{i-1}=Trans_{z,-d_{i}}Rot_{z,-\theta_{i}}Trans_{x,-a_{i-1}}Rot_{x,-\alpha_{i-1}}$$

where $Rot_{L,\psi }$ and $Trans_{L,r}$ corresponds to the homogeneous transformation matrices rotated by angle ψ about axis *L* and translated by distance *r* along axis *L* [27], respectively, while $d_{i}$, $\alpha_{i}﻿$, and $\theta_{i}$ are listed in Figure 3 and Table 1. ${}^{j}T_{i}\left(i\neq j\right)$ is also expressed as:

(3) $$\begin{array}{c} {}^{j}T_{i}= \end{array}\left[\begin{array}{cc} {}^{j}R_{i} & {}^{j}P_{i}\\ 0 & 1 \end{array}\right]\begin{array}{c} = \end{array}\left[\begin{array}{cccc} {}^{j}n_{i} & {}^{j}o_{i} & {}^{j}a_{i} & {}^{j}P_{i}\\ 0 & 0 & 0 & 1 \end{array}\right]$$

where ${{}^{j}R}_{i}$ and ${}^{j}P_{i}$ represent the rotation matrix and position vector, respectively.

### 2.3. Inverse Kinematics

In this paper, an additional constraint is introduced to restrain the self-motion of SSRMS-type manipulators that aligns axis $z_{2}$ (i.e., $y_{1}$) with axis $z_{6}$ (i.e., $y_{5}$), so that the finite possible poses of the manipulator are determinate and the corresponding inverse kinematics solutions are easy to obtain according to the desired ${{}^{0}T}_{7}$.

#### 2.3.1. Analytical Solution of $\theta_{1}$ and $\theta_{7}$

Restrained by the alignment constraint, axis $y_{1}$ must be parallel to the intersection line of plane $O_{0}xy$ and $O_{7}xy$. For the desired ${}^{0}T_{7}$, the equation of plane $O_{7}xy$ can be expressed by the position of $O_{7}$ (namely ${}^{0}P_{7}$) and the normal vector $z_{7}$ (namely ${}^{0}a_{7}$), and the equation of plane $O_{1}xy$ can be expressed as $z=d_{1}$. Combining the above two plane equations gives the equations of the family of lines parallel to $y_{1}$ expressed in the base coordinate $\sum_{0}$:

(4) $${{}^{0}a}_{7}^{x}x+{{}^{0}a}_{7}^{y}y=Constant$$

Considering the direction of $y_{1}$, two solutions for the first joint angle $\theta_{1}$, that is the angle from $x_{0}$ to $x_{1}$ measured about $z_{1}$, are obtained

(5) $$\begin{array}{c} \theta_{1}\left(1\right)=atan2\left({{}^{0}a}_{7}^{y},{{}^{0}a}_{7}^{x}\right)\\ \theta_{1}\left(2\right)=atan2\left(-{{}^{0}a}_{7}^{y},-{{}^{0}a}_{7}^{x}\right) \end{array}$$

where the four-quadrant arctangent function atan2(y,x) returns the angle from the origin to (x,y) in the plane in the range (−π,π].

For the convenience of expression and programming, Equation (5) is rewritten as

(6) $$\begin{array}{c} \theta_{1}\left(1\right)=atan2\left({{}^{0}T}_{7}\left(2,3\right),{{}^{0}T}_{7}\left(1,3\right)\right)\\ \theta_{1}\left(2\right)=atan2\left(-{{}^{0}T}_{7}\left(2,3\right),-{{}^{0}T}_{7}\left(1,3\right)\right) \end{array}$$

In analogy to the above, the angle rotating from $x_{7}$ to $x_{6}$ about $z_{7}$, i.e.,$-\theta_{7}$, can be obtained according to the specified ${}^{7}T_{0}$. Hence, two solutions for the seventh joint angle $\theta_{7}$ can be computed:

(7) $$\begin{array}{c} \theta_{7}\left(1\right)=-atan2\left(-{{}^{0}T}_{7}\left(3,2\right),-{{}^{0}T}_{7}\left(3,1\right)\right)\\ \theta_{7}\left(2\right)=-atan2\left({{}^{0}T}_{7}\left(3,2\right),{{}^{0}T}_{7}\left(3,1\right)\right) \end{array}$$

It exploits the fact about the rotation matrix ${{}^{7}R}_{0}={{}^{0}R}_{7}^{T}$ to avoid explicitly computing ${}^{7}T_{0}$.

It can be seen that four solutions exist depending on the values of $\theta_{1}$ and $\theta_{7}$. The solutions $\left(\theta_{1}\left(1\right),\theta_{7}\left(1\right)\right),\left(\theta_{1}\left(2\right),\theta_{7}\left(2\right)\right)$ are relative to $z_{2}=z_{6}$, while $\left(\theta_{1}\left(1\right),\theta_{7}\left(2\right)\right),\left(\theta_{1}\left(2\right),\theta_{7}\left(1\right)\right)$ are for $z_{2}={-z}_{6}$.

#### 2.3.2. Analytical Solution of $\theta_{2}$ and $\theta_{6}$

Upon determining $\theta_{1}$ and $\theta_{7}$, considering the matrix equation ${{}^{0}T}_{7}={{}^{0}T}_{1}{{}^{1}T}_{2^{\prime }}{{}^{2^{\prime }}T}_{6}{{}^{6}T}_{7}$, then ${}^{2^{\prime }}T_{6}$ can be calculated using the property ${}^{i}T{}_{j}^{-1}={{}^{j}T}_{i}$.

(8) $${{}^{2^{\prime }}T}_{6}={{}^{2^{\prime }}T}_{1}{{}^{1}T}_{0}{{}^{0}T}_{7}{{}^{7}T}_{6}$$

where

(9) $${}^{2^{\prime }}T_{1}=Trans_{z,-d_{2}}Rot_{x,-\alpha_{1}}$$

**Figure 4:**
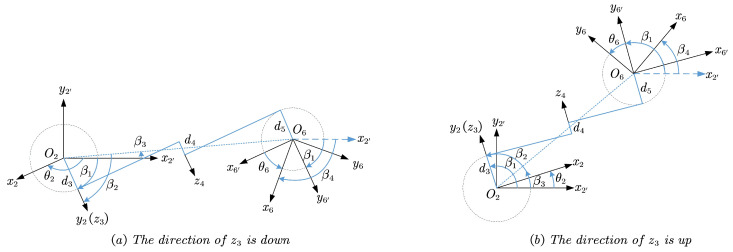
Solving $\theta_{2}$ and $\theta_{6}$ when $z_{2}$ and $z_{6}$ have the same orientation.

Projecting the axes of $\sum_{i}\left(i=2,2^{\prime },6,6^{\prime }\right)$ onto plane $O_{2}xy$ along the opposite direction of $z_{2}$, the geometric relationship between them is shown in Figure 4 when $z_{2}$ and $z_{6}$ are in the same direction. As illustrated, depending on whether the direction of $z_{3}$ is up or down, there exists two possible poses, corresponding to each condition.

Let $\beta_{1}$ be the angle from $x_{2^{\prime }}$ to $y_{2}$, $\beta_{1}$ be from the projection of $O_{2}O_{6}$ to $y_{2}$, and $\beta_{3}$ be from $x_{2^{\prime }}$ to the projection of $O_{2}O_{6}$, which are measured around $z_{2}$.

(10) $$\beta_{2}=arccos\left(\frac{\left|d_{3}+d_{4}+d_{5}\right|}{\sqrt{{}^{2^{\prime }}T_{6}{\left(1,4\right)}^{2}+{}^{2^{\prime }}T_{6}{\left(2,4\right)}^{2}}}\right)$$

(11) $$\beta_{3}=atan2\left({{}^{2^{\prime }}T}_{6}\left(2,4\right),{{}^{2^{\prime }}T}_{6}\left(1,4\right)\right)$$

According to the geometric relations depicted in Figure 4, the quantitative relations between $\beta_{2}$, $\beta_{3}$, and $\beta_{4}$ can be expressed as follows.

(12) $$\beta_{1}=\left\{\begin{array}{cc} +\beta_{2}+\beta_{3} & \mathrm{if}\;z_{3}\;is\;down\\ -\beta_{2}+\beta_{3} & \mathrm{if}\;z_{3}\;is\;up \end{array}\right.$$

Equation (10) to Equation (12) stay the same when $z_{2}$ and $z_{6}$ are in the opposite directions.

Considering the difference between the definition of $\theta_{2}$ and $\beta_{2}$, $\theta_{2}$ can be obtained:

(13) $$\theta_{2}=\beta_{1}-\pi /2$$

Namely

(14) $$\begin{array}{c} \theta_{2}\left(1\right)=+\beta_{2}+\beta_{3}-\pi /2\\ \theta_{2}\left(2\right)=-\beta_{2}+\beta_{3}-\pi /2 \end{array}$$

It is worth noting that $\theta_{1}$ is not related to the direction of $z_{6}$ while $\theta_{6}$ is related. Similar to the previous, let $\beta_{4}$ be the angle from $x_{2^{\prime }}$ to $x_{6}$ around $z_{2}$, note that it is not a rotation around $z_{6}$. Hence, $\theta_{6}$ can be represented by $\beta_{4}$ and $\beta_{1}$:

(15) $$\theta_{6}=\left\{\begin{array}{cc} \pi /2+(\beta_{4}-\beta_{1}) & \mathrm{if}\;z_{2}=z_{6}\\ \pi /2-(\beta_{4}-\beta_{1}) & \mathrm{if}\;z_{2}=-z_{6} \end{array}\right.$$

Considering that $\theta_{6}$ is computed from the given ${{}^{2^{\prime }}T}_{6}$, that is, the relationship between $z_{2}$ and $z_{6}$ depends on the actual value of ${{}^{2^{\prime }}T}_{6}$, in particular on the sign of ${{}^{2^{\prime }}T}_{6}\left(3,3\right)$ that is equal to 1 when $z_{2}=z_{6}$ and equal to −1 when $z_{2}=-z_{6}$. Therefore, substituting Equation (12) for the Equation (15), and rewriting Equation (15) using the information provided by ${{}^{2^{\prime }}T}_{6}\left(3,3\right)$ for uniformity of expression and ease of programming, we obtain

(16) $$\begin{array}{c} \theta_{6}\left(1\right)=\pi /2+sign\left({{}^{2^{\prime }}T}_{6}\left(3,3\right)\right)\left(\beta_{4}-\beta_{2}-\beta_{3}\right)\\ \theta_{6}\left(2\right)=\pi /2+sign\left({{}^{2^{\prime }}T}_{6}\left(3,3\right)\right)\left(\beta_{4}+\beta_{2}-\beta_{3}\right) \end{array}$$

Now, for each input $\left(\theta_{1},\theta_{7}\right)$, two analytical solutions for $\theta_{2}$ and $\theta_{6}$ are obtained, namely $\left(\theta_{1}\left(1\right),\theta_{6}\left(1\right)\right)$ and $\left(\theta_{1}\left(2\right),\theta_{6}\left(2\right)\right)$.

#### 2.3.3. Analytical Solution of $\theta_{3}$, $\theta_{4}$, and $\theta_{5}$

Similarly, having $\theta_{i}\left(i=1,2,6,7\right)$, considering the matrix equation ${{}^{2^{\prime }}T}_{6}={{}^{2^{\prime }}T}_{2}{{}^{2}T}_{3^{\prime }}{{}^{3^{\prime }}T}_{5}{{}^{5}T}_{6}$, then ${}^{3^{\prime }}T_{5}$ can be calculated

(17) $${{}^{3^{\prime }}T}_{5}={{}^{3^{\prime }}T}_{2}{{}^{2}T}_{2^{\prime }}{{}^{2^{\prime }}T}_{6}{{}^{6}T}_{5}$$

where,

(18) $$\begin{array}{c} {{}^{3^{\prime }}T}_{2}=Trans_{z,-d_{3}}Rot_{x,-\alpha_{2}}\\ {{}^{2}T}_{2^{\prime }}=Rot_{z,-\theta_{2}} \end{array}$$

**Figure 5:**
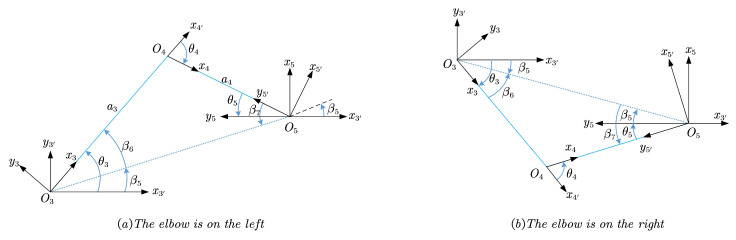
Solving $\theta_{3}$, $\theta_{4}$, and $\theta_{5}$ when $z_{2}$ and $z_{6}$ have the same orientation.

Projecting the axes of $\sum_{i}\left(i=3,3^{\prime },5,5^{\prime }\right)$ onto plane $O_{3}xy$ along the opposite direction of $z_{3}$, the geometric relationship between them is shown in Figure 5 when $z_{2}$ and $z_{6}$ point in the same direction. As shown in the figure, depending on whether the pose of the elbow is on the left or right, two admissible configurations exist corresponding to each condition.

Let $\beta_{5}$ be the angle from $x_{3^{\prime }}$ to the projection of $O_{3}O_{5}$, $\beta_{6}$ be from $x_{3}$ to the projection of $O_{3}O_{5}$, and $\beta_{7}$ be from $x_{4}$ to the projection of $O_{3}O_{5}$, all of them are measured around $z_{3}$.

(19) $$\beta_{5}=atan2\left({{}^{3^{\prime }}T}_{5}\left(2,4\right),{{}^{3^{\prime }}T}_{5}\left(1,4\right)\right)$$

(20) $$\beta_{6}=arccos\left(\frac{a_{3}^{2}+{{}^{3^{\prime }}T}_{5}{\left(1,4\right)}^{2}+{{}^{3^{\prime }}T}_{5}{\left(2,4\right)}^{2}-a_{4}^{2}}{2a_{3}\sqrt{{{}^{3^{\prime }}T}_{5}{\left(1,4\right)}^{2}+{{}^{3^{\prime }}T}_{5}{\left(2,4\right)}^{2}}}\right)$$

(21) $$\beta_{7}=arccos\left(\frac{a_{4}^{2}+{{}^{3^{\prime }}T}_{5}{\left(1,4\right)}^{2}+{{}^{3^{\prime }}T}_{5}{\left(2,4\right)}^{2}-a_{3}^{2}}{2a_{4}\sqrt{{{}^{3^{\prime }}T}_{5}{\left(1,4\right)}^{2}+{{}^{3^{\prime }}T}_{5}{\left(2,4\right)}^{2}}}\right)$$

According to the geometric relations shown in Figure 5 where $z_{2}$ and $z_{6}$ are orientated in the same direction, the quantitative relation between $\beta_{5}$, $\beta_{6}$, and $\beta_{7}$ can be expressed as follows

(22) $$\theta_{3}=\beta_{5}\pm \beta_{6}$$

(23) $$\theta_{4}=\pm \left(\beta_{6}+\beta_{7}\right)$$

(24) $$\theta_{5}=\left\{\begin{array}{cc} -\beta_{5}\pm \beta_{7} & \mathrm{if}\;z_{2}=z_{6}\\ -\beta_{5}\pm \beta_{7}+\pi & \mathrm{if}\;z_{2}=-z_{6} \end{array}\right.$$

Equation (22) to Equation (24) stay the same when $z_{2}$ and $z_{6}$ are in opposite directions, and the pending positive and negative signs depend on the posture of the elbow, while whether $z_{2}$ and $z_{6}$ point in the same direction can be obtained by the value of ${{}^{3^{\prime }}T}_{5}\left(3,3\right)$, as in the case of solving $\theta_{6}$. Rewriting Equations (22) to (24) yields two acceptable solutions from $\theta_{3}$ to $\theta_{5}$ for each input $\left(\theta_{1},\theta_{2},\theta_{6},\theta_{7}\right)$:

(25) $$\begin{array}{c} \theta_{3}\left(1\right)=\beta_{5}+\beta_{6}\\ \theta_{4}\left(1\right)=-\left(\beta_{6}+\beta_{7}\right)\\ \theta_{5}\left(1\right)=-\beta_{5}+\beta_{7}+\frac{1-{{}^{3^{\prime }}T}_{5}\left(3,3\right)}{2}\pi \end{array}$$

(26) $$\begin{array}{c} \theta_{3}\left(2\right)=\beta_{5}-\beta_{6}\\ \theta_{4}\left(2\right)=\beta_{6}+\beta_{7}\\ \theta_{5}\left(2\right)=-\beta_{5}-\beta_{7}+\frac{1-{{}^{3^{\prime }}T}_{5}\left(3,3\right)}{2}\pi \end{array}$$

#### 2.3.4. Sixteen Solutions

Upon obtaining the above, sixteen analytical solutions for inverse kinematics of SSRMS-type manipulators are obtained simultaneously, taking into account four geometric features. These factors include whether $z_{2}$ and $z_{6}$ are in the same direction, whether the angle between $z_{1}$ and $z_{7}$ is acute or obtuse, whether the direction of $z_{3}$ is up or down (as shown in Figure 4), and whether the posture of the elbow is on the left or right (as shown in Figure 5). Each of these factors resulted in two possible postures and were combined to obtain the sixteen solutions listed in Table 2, where the values indicate the $\theta_{i}$(i=1,2,…,7) index of each solution. For SSRMS-type manipulators, the posture of the elbow plays a significant role in determining the manipulators’ configuration and can be determined using an obstacle avoidance algorithm. The other three factors can be used to prevent joint limits.

## 3. Algorithm Analysis

### 3.1. Singularity of the Proposed Method

All of the obtained solutions are based on the existence of an intersection line between $O_{0}xy$ and $O_{7}xy$. Therefore, the above algorithm is singular when $O_{0}xy$ and $O_{7}xy$ are parallel, i.e., when $z_{0}$ and $z_{7}$ are in the same or opposite directions. Specifically, $\theta_{1}$ and $\theta_{7}$ are under-constrained when the singularity occurs. Thus, $\theta_{1}$ can be specified arbitrarily and the alignment constraint is satisfied by the value of $\theta_{7}$ and vice versa, which is determined by

(27) $$\theta_{7}+\theta_{1}=\gamma$$

where γ is an angle variable calculated from ${}^{0}T_{7}$

(28) $$\gamma =sign\left({}^{0}T_{7}\left(3,3\right)\right)\times sign\left({}^{0}T_{7}\left(2,1\right)\right)\times arccos\left({}^{0}T_{7}\left(1,1\right)\right)$$

The term $sign({}^{0}T_{7}\left(3,3\right))$ indicates whether $z_{0}$ and $z_{7}$ are oriented in the same direction, and $sign\left({}^{0}T_{7}\left(2,1\right)\right)\times arccos\left({}^{0}T_{7}\left(1,1\right)\right)$ corresponds to the rotation angle from $x_{0}$ to $x_{7}$ around $z_{0}$.

Considering the physical meaning of the alignment constraint, once $\left(\theta_{1},\theta_{7}\right)$ satisfies Equation (27), then, $\left(\theta_{1},\theta_{7}-\pi \right)$, $\left(\theta_{1}-\pi ,\theta_{7}\right)$, and $\left(\theta_{1}-\pi ,\theta_{7}-\pi \right)$ are also acceptable.

In addition, for planning the trajectory through the aforementioned singular configurations, $\theta_{1}$ and $\theta_{7}$ can be computed from the joint angle interpolation from the preceding and following moments to smooth the trajectory in joint space.

### 3.2. Unsolvable Poses

In the casing of solving $\theta_{2}$ and $\theta_{6}$, the existence of $\beta_{3}$ obviously implies that

(29) $$\left|d_{3}+d_{4}+d_{5}\right|<\sqrt{{}^{2^{\prime }}T_{6}{\left(1,4\right)}^{2}+{}^{2^{\prime }}T_{6}{\left(2,4\right)}^{2}}$$

and when computing from $\theta_{3}$ to $\theta_{5}$, the acceptable solutions for $\beta_{6}$ and $\beta_{7}$ require that

(30) $$\sqrt{{{}^{3^{\prime }}T}_{5}{\left(1,4\right)}^{2}+{{}^{3^{\prime }}T}_{5}{\left(2,4\right)}^{2}}<a_{3}+a_{4}$$

Unfortunately, neither Equation (29) nor Equation (30) can be strictly guaranteed by the above method. Two kinds of representative unsolvable poses can be identified through intuition, that is, the end-effector (i.e., $O_{7}$) is too close or too far from the base (i.e., $O_{0}$) when a possible $\theta_{3}$ is close to π or −π, respectively. That is to say, the workspace of the SSRMS-type manipulator is reduced by the proposed alignment constraint such that some real possible poses cannot be resolved using the above method.

Fortunately, the reduction in the workspace is relatively small, as illustrated by the statistics of the simulation results later. More meaningful is the fact that manipulators generally operate beyond such reduced spaces and this problem did not occur on the SRS-type manipulators.

## 4. Verification and Simulation

### 4.1. Efficiency and Accuracy

The efficiency and accuracy of the proposed inverse kinematic solutions above and the ikine function in the Robotics Toolbox for MATLAB@(RTB-M) by Peter Corke [28] are investigated and compared. Of course, comparing analytical and optimization methods using metrics, such as efficiency, is unreasonable and unfair. In this paper, we intend to use an objective and reproducible third-party algorithm as a baseline to evaluate the performance of the proposed approach, just like RTB-M.

Two forward kinematics models for SSRMS-type and SRS-type manipulators, respectively, were established with RTB-M using the DH parameters listed in Table 1. Both manipulators are arranged in the (R–Y–P)–P–(P–Y–R) configuration, and the difference is whether the offset variables $d_{3}$ and $d_{6}$ are zero or not. Then, a total of 10,000 sets of joint angles with a uniform distribution ranging from −π to π were randomly generated using the generator function rng(2,‘philox’) embedded in MATLAB@ and were converted to 10,000 corresponding to the desired ${}^{0}T_{7}$ by the forward kinematics models. At last, both the proposed approach and function $ikine({}^{0}T_{7},‘tol’,$ $1\times 10^{-11})$ were used to solve the inverse kinematics for those given ${}^{0}T_{7}$, and the statistics of the simulation results are listed in Table 3.

As shown in Table 3, both methods were evaluated in terms of average calculation time, success rate, and average position error. It should be noted that parameter ‘tol’ has very little effect on the calculation time of the ikine function when ‘tol’ is relatively small. According to the simulation results, the following conclusions can be drawn:

1. The average calculation time of the proposed method and RTB-M for the SSRMS-type manipulator with approximate position errors are 0.133 ms and 21.26 ms, respectively, and 0.297 ms and 27.2 ms for the SRS-type manipulator, respectively. The results show that the computational efficiency of the proposed method is two orders of magnitude faster than RTB-M;
2. The success rates of the proposed method for SSRMS-type and SRS-type manipulators are 92.29% and 100%, respectively, both are significantly greater than RTB-M. When used to solve the inverse kinematics for SSRMS-type manipulators, the proposed method suffers from the reduction in workspace caused by the alignment constraint and cannot obtain the solutions of some real possible poses.

### 4.2. Unsolvable Poses

**Figure 6:**
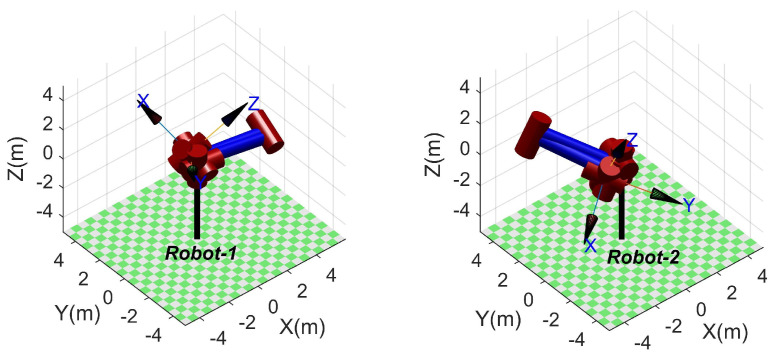
Examples of unsolvable poses: θ=[1.43,−1.22,0.10,3.08,0.82,−1.53,−2.40] (for **left**) and θ=[2.93,−2.68,−0.97,−3.03,3.09,2.53,0.08] (for **right**).

**Figure 7:**
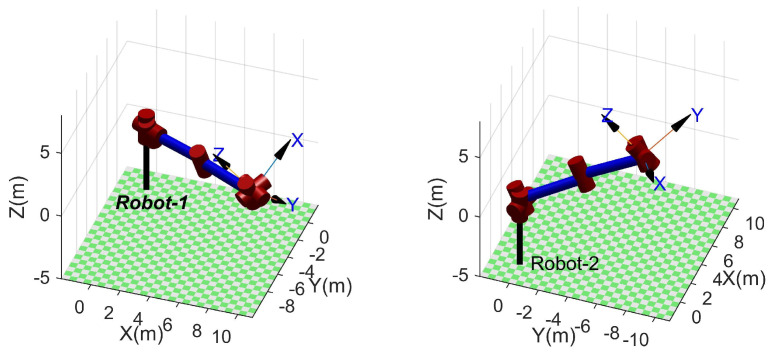
Examples of unsolvable poses: θ=[1.08,2.79,−0.06,0.05,−1.45,1.69,−2.06] (for **left**) and θ=[1.37,−0.46,−1.07,−0.13,−1.00,0.33,−1.77] (for **right**).

The proposed method cannot solve some practically possible poses when used to solve the inverse kinematic of an SSRMS-type manipulator subjected to the reduction in workspace caused by itself. Two kinds of representative unsolvable poses can be identified by intuition, that is, the end-effector is too close or too far from the base when a possible $\theta_{3}$ is close to π or −π. Two unsolvable poses for each case are visualized in Figure 6 and Figure 7 using RTB-M. Frustratingly, the proposed method cannot give the range of unsolvable poses strictly, although it can recognize them using Equations (29) and (30).

From a statistical point of view, the success rate of the proposed method is 92.29% and 100% for SSRMS-type and SRS-type manipulators, respectively. That is, the reduction in the workspace is relatively small. Moreover, according to Equations (29) and (30), two kinds of representative unsolvable poses can be identified through intuition, that is, the end-effector (i.e., $O_{7}$) is too close or too far from the base (i.e., $O_{0}$) when a possible $\theta_{3}$ is close to π or −π, respectively. It is worth pointing out that manipulators generally operate outside of this reduced space and this problem does not occur with the SRS-type manipulators.

### 4.3. Singularity Problem

Yang et al. [2] pointed out that those methods based on arm angle parameterization are singular when the reference vector and the wrist point vector are collinear, and solve this problem via the dual arm angle parameterization method. Jin’s method [25] encounters a singularity problem when $z_{2}=\pm z_{6}$ and adopts an optimization method to determine the joint angles, and coincidentally, the proposed method can cope with such situations. Nokleby [4] obtained four singular configurations of SSRMS using screw theory, and the second one corresponds to singular configurations of the presented method.

All of the mentioned singularity problems can be solved using the proposed method, no matter if it is caused by the method itself or the singular configuration of the SSRMS. For the sake of simplicity, this paper lists the solutions for an example (θ = [0°, 0°, 45°, 125°, 45°, 0°, 0°]) of the second singular configuration given by [4] . The corresponding desired homogeneous transformation matrix ${}^{0}T_{7}^{*}$ is computed with DH parameters listed in Table 1.

(31) $$\begin{array}{c} {}^{0}T_{7}^{*}= \end{array}\left[\begin{array}{cccc} -0.8192 & 0.5736 & 0 & 3.7032\\ -0.5736 & -0.8192 & 0 & 1.1676\\ 0 & 0 & 1 & 0.9000\\ 0 & 0 & 0 & 1 \end{array}\right]$$

The value γ is calculated from Equation (27) as −2.5307. An acceptable solution [0.6283, −2.6622, −0.2685, −2.2646, 2.5331, 2.6622, 3.1241] is obtained using the proposed method, where $\theta_{1}$ is randomly set to 0.6283.

### 4.4. Planning Trajectory with Singular Configurations

**Figure 8:**
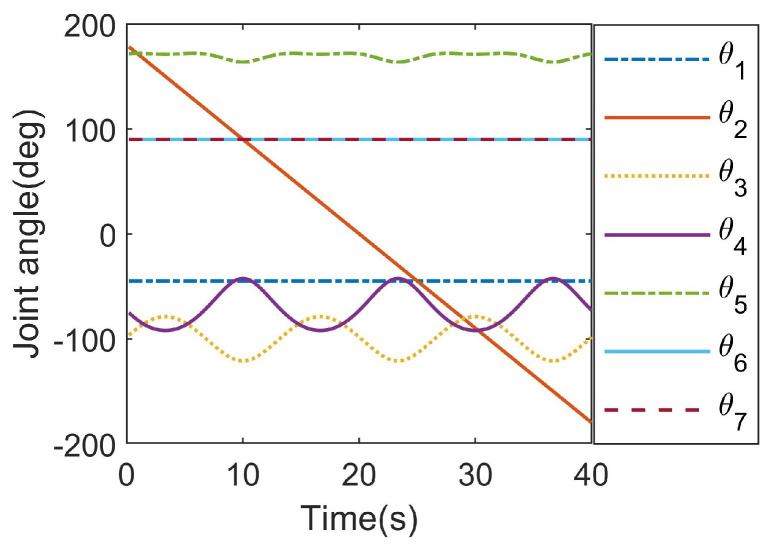
The planned joint angle curves.

**Figure 9:**
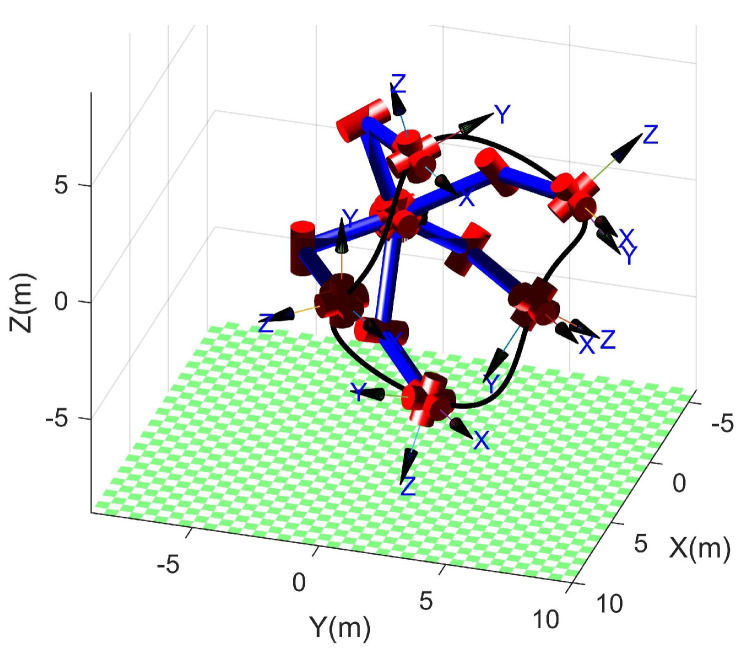
The desired trajectory and some of the planned poses.

To verify the ability of the proposed method for planning the trajectory with singular configurations, an experimental task was set in which the end effector tracked a desired trajectory that was a superposition of a circle and an out-of-plane sinusoidal offset with an amplitude of 0.75 m. The circle has a center at (3.58, 3.58, 0) (m) and a radius of 5 m, and is located in the plane that is parallel to $z_{0}$ and 45 degrees to both $x_{0}$ and $y_{0}$. The desired direction of $x_{7}$ is normal to the plane and the direction of $z_{7}$ is along the radial direction of the circle and away from the center. In this case, the proposed method encountered a singularity problem when the end effector moved to the top and the bottom of the $z_{0}$ direction. As a result, the planned joint angle curves shown in Figure 8 are continuous and vary smoothly, and the desired trajectory (black line) and some planned poses are visualized in Figure 9 using RTB-M.

## 5. Conclusions

Herein, an analytical solution was proposed to solve the inverse kinematics of SSRMS-type redundant manipulators based on an alignment constraint between the axes of the second and sixth joints, and this was obtained by dealing with the SSRMS-type manipulator directly instead of a simplified SRS-type manipulator. The constraint introduced here is added automatically rather than manually or by other algorithms and decomposes the spatial inverse kinematics problem into three independent planar subproblems. The resulting geometric equations concerning part of the joint angles could be computed recursively and efficiently using the sequences of $\left(\theta_{1},\theta_{7}\right)$, $\left(\theta_{2},\theta_{6}\right)$, and $\left(\theta_{3},\theta_{4},\theta_{5}\right)$, generating up to 16 sets of solutions for a given desired end-effector pose. Additionally, the singularity problem of the proposed approach was figured out and solved using a complementary method, and unsolvable desired poses were discussed. A numerical simulation was conducted to investigate the performance of the proposed approach in terms of average calculation time, success rate, average position error, and planning trajectory with singular configurations, and to verify the ability to avoid singularities and to recognize unsolvable poses. The simulation results show that the proposed method performs better than RTB-M on SSRMS-type and SRS-type manipulators, and the proposed method suffers from the reduction in the workspace, a problem that does not occur on the SRS-type manipulators.

## Figures and Tables

**Table 1:** The modified DH parameters of the SSRMS-type manipulator.

i	$\alpha_{i-1}$ (deg)	$a_{i-1}$ (m)	$d_{i}$ (m)	$\alpha_{i}$ (deg)	offset (deg)
1	0	0	$d_{1}=0.65$	$\theta_{1}$	0
2	$\alpha_{1}=90$	0	$d_{2}=$ 0.3	$\theta_{2}$	0
3	$\alpha_{2}=-90$	0	$d_{3}=$ 0.3	$\theta_{3}$	−90
4	0	$\;a_{3}=$ 4.4	$d_{4}=$ 0.3	$\theta_{4}$	0
5	0	$a_{4}=$ 4.4	$d_{5}=$ 0.3	$\theta_{5}$	90
6	$\alpha_{5}=90$	0	$d_{6}=$ 0.3	$\theta_{6}$	0
7	$\alpha_{6}=-90$	0	$d_{7}=$ 0.65	$\theta_{7}$	0

**Table 2:** Sixteen sets of IK solutions of SSRMS-type manipulators.

Solution	$\theta_{1}$	$\theta_{2}$	$\theta_{3}$	$\theta_{4}$	$\theta_{5}$	$\theta_{6}$	$\theta_{7}$
No. 1	1	1	1	1	1	1	1
No. 2	1	1	2	2	2	1	1
No. 3	1	2	1	1	1	2	1
No. 4	1	2	2	2	2	2	1
No. 5	1	1	1	1	1	1	2
No. 6	1	1	2	2	2	1	2
No. 7	1	2	1	1	1	2	2
No. 8	1	2	2	2	2	2	2
No. 9	2	1	1	1	1	1	1
No. 10	2	1	2	2	2	1	1
No. 11	2	2	1	1	1	2	1
No. 12	2	2	2	2	2	2	1
No. 13	2	1	1	1	1	1	2
No. 14	2	1	2	2	2	1	2
No. 15	2	2	1	1	1	2	2
No. 16	2	2	2	2	2	2	2

**Table 3:** Performance of the proposed method and RTB-M.

Manipulator	Method	Average Calculation Time (ms)	Success Rate (%)	Average Position Error (mm)
SSRMS-type	The proposed method	0.133	92.29	3.4 $\times \;10^{-12}$
	RTB-M	21.26	84.19	1.97 $\times \;10^{-12}$
SRS-type	The proposed method	0.297	100	5.7 $\times \;10^{-12}$
	RTB-M	27.2	84.19	1.7 $\times \;10^{-12}$

## Data Availability

No new data were created.
